# Trajectories of Depressive Symptoms and Associated Risk Factors From Late Adolescence to Emerging Adulthood

**DOI:** 10.32872/cpe.15801

**Published:** 2025-11-28

**Authors:** Simone Pfeiffer, Philipp Alt, Sabine Walper

**Affiliations:** 1 Clinical Child and Adolescent Psychology and Psychotherapy, RPTU Kaiserslautern-Landau, Landau, Germany; 2 Department of Education and Rehabilitation, Ludwig-Maximilians University of Munich, Munich, Germany; 3 German Youth Institute (DJI), Munich, Germany; Philipps-University of Marburg, Marburg, Germany

**Keywords:** depressive symptom trajectories, adolescents, emerging adulthood, risk factors

## Abstract

**Background:**

This study addresses a research gap by identifying depressive symptom trajectories from adolescence to emerging adulthood in a German community sample using a person-centered approach.

**Method:**

The sample consisted of 3,682 adolescents and young adults (49.3% self-identified as female; age at T1: 15–19 years, *M* = 17.03, *SD* = 0.88) assessed in seven annual waves of the German Family Panel Pairfam. Latent class growth analysis was conducted with sociodemographic variables (gender, family status, parental education, economic deprivation, immigration background) and depressive symptoms, as assessed by the State-Trait Depression Scales.

**Results:**

Five depressive symptom trajectories were identified: stable low symptoms (34%), intermediate onset with decreasing symptom trajectory (8%), intermediate onset with slow increasing symptom trajectory (46%), intermediate onset with strong increase symptom trajectory (9%) and stable high symptoms (4%). Female gender and economic deprivation were predictors for all four classes associated with higher depressive symptoms with reference to the class with stable low depressive symptoms. Family status and immigration status lost their predictive impact for membership in depressive symptom trajectories when economic deprivation was included.

**Conclusions:**

Interventions should target the underlying etiological factors of female gender and economic deprivation being risk factors for trajectories of depression, taking into consideration the complexity and interaction of biopsychosocial and political variables in the development of depressive disorders.

## Highlights

- Five depressive symptom trajectories were identified from adolescence to emerging adulthood (15-25 years) in a German sample.
- Female gender and economic deprivation are risk factors and should be targeted in preventions measures for depression.
- Economic deprivation mediates the effects of family status and immigration status on depressive symptoms.

Emerging adulthood is considered a distinct period between the ages of 18 and 25 years (Arnett, 2000) in which the successful handling and accomplishments of developmental milestones is associated with the transition into adulthood. The conditions under which individuals cope with these developmental milestones serve as risk factors for the development of mental health problems (Baggio et al., 2017; Ladhani et al., 2019). The onset of depression increases significantly in this period with a peak at the age of 15–17 years (Schubert et al., 2017) with prevalence rates of depressive disorders at 3–18% (Kessler et al., 2012; Polanczyk et al., 2015). After the peak in adolescence, depressive symptoms tend to decrease and seem to remain relatively stable during adulthood (Salk et al., 2017). However, these overall trends do not reflect individual trajectories, which may comprise stable courses of high or low depressiveness as well as increases or recoveries. Identifying types of detrimental trajectories, their prevalence, and their related risk factors can provide important information for health services and the development of targeted prevention programs.

Understanding risk and protective factors in the development of depressive disorders is highly relevant, as untreated depressive disorders in adolescence are associated with, among other outcomes, the development of chronic depressive symptoms, suicide, substance abuse, lower educational outcome, and lower income as an adult (Chen & Kaplan, 2003; Hawton et al., 2012).

This is the first study investigating trajectories of depressive symptoms from adolescence to emerging adulthood in a German community sample, where mental disorder treatment is covered by mandatory health insurance. Accordingly, by taking into account dimensional indicators of depressive symptoms and adopting a person-centered approach, we aimed to identify trajectories of depressive symptoms from adolescence to young adulthood and to investigate sociodemographic risk factors for detrimental trajectories.

## Depressive Symptom Trajectories From Adolescence to Emerging Adulthood

Interindividual differences in the dynamics of symptom development over time are best captured by person-centered approaches, which reflect the heterogeneity of symptom development across time. In a systematic review focusing on trajectory studies of depressive symptoms in late adolescence and emerging adulthood (15–24 years), the vast majority of studies found three to four trajectories, with a lower number of trajectories being more likely among younger adolescents (Schubert et al., 2017). In most studies, 40–55% of the assessed subjects fell into the trajectories with stable low or no depressive symptoms (stable low/absence of symptoms). A second reoccurring trajectory was found for a group of subjects (5–15%) with constantly high depressive symptoms (persistent depression/stable high). Furthermore, there was at least one group with less severe and less stable symptoms with varying increasing or decreasing slopes over time. In these intermediate trajectories external factors might play a more important role compared to the trajectories with low and high persistent symptoms (Schubert et al., 2017). Only a few studies investigated trajectories of depressive symptoms from adolescence to emerging adulthood in European samples, where mental disorder treatment is often covered by mandatory public health insurance. Evidence for depressive symptom trajectories has been reported for samples in the United Kingdom (Gaysina et al., 2011; St Clair et al., 2012), the Netherlands (Diamantopoulou et al., 2011; Lubke et al., 2016; Maciejewski et al., 2019), and Sweden (Lallukka et al., 2019).

It is known that specific demographic factors are associated with higher depressive burden. The identification of risk and protective factors might be useful for understanding the etiological factors in different symptom trajectories. Female adolescents, for example, have a higher incidence of major depression, a more chronic trajectory, an earlier age of onset, and a higher increase in depressive symptoms compared to male adolescents (Crockett et al., 2020; Edwards et al., 2014; Hargrove et al., 2020; Mezulis et al., 2014). In a meta-analysis of depressive symptom trajectories in children and adolescents, high or increasing depressive symptom trajectories were also predominantly predicted by female gender (Shore et al., 2018). There is evidence for a decrease of gender differences in emerging adulthood and a stabilization during adulthood (Salk et al., 2017).

Other predictors of increased depressive symptoms in adolescence and young adulthood include lower parental income and poverty, often preceded by lower parental education (Korhonen et al., 2017; Najman et al., 2010; Quesnel-Vallée & Taylor, 2012; Wickrama et al., 2009). However, evidence suggests stronger associations between socioeconomic status and externalizing problems compared to internalizing problems. When family income increased, externalizing symptoms of children (e.g., aggressive behavior) seemed to decrease, but internalizing symptoms persisted (Costello et al., 2003). Furthermore, cross-sectional and longitudinal findings suggest that female adolescents may be more vulnerable to economic deprivation than their male peers regarding their depressiveness (Walper, 2009). Higher education, being associated with higher family income, may also be a protective factor (Mendolicchio & Rhein, 2011).

Family type has also been shown to matter, with results indicating higher depressive symptoms among young people from single-parent households (Björkenstam et al., 2017; Laukkanen et al., 2016; Sieh et al., 2013), whereas living in a two-parent household was identified as a protective factor for developing a depressive disorder (Costello et al., 2008). However, stepfamilies can bear increased risks of children’s and adolescents’ adjustment problems (Jeynes, 2006). Increased stress in the context of parental separation and/or stepfamily formation and compromised family relationships may contribute to a higher risk of depression (Jensen & Lippold, 2018; Shafer et al., 2017).

Finally, there is evidence that adolescents with an immigration background and those who belong to an ethnic minority experience higher levels of depressive symptoms and an earlier age of onset compared to people with a nonimmigrant background, not only in U.S. samples (Brown et al., 2007; Costello et al., 2008), but also in European samples (Belhadj Kouider et al., 2014). An immigration background and membership in an ethnic minority is considered a risk factor for experiencing acculturation distress, facing discrimination, racism, and struggles in the process of identity development, which are, in turn, associated with an increased risk of the development of depressive symptoms (Desalu et al., 2019; Meca et al., 2019). We sought to replicate these findings in the present study.

## Current Study

This is the first study investigating trajectories of depressive symptoms from adolescence to emerging adulthood in a German community sample. The first aim of this study was to investigate whether subgroups of adolescents transitioning into young adulthood differ in their course of depressive symptoms, using longitudinal data drawn from seven annual waves of the German Family Panel (pairfam). Taking prior research (Schubert et al., 2017) into consideration, we expected to identify four to six trajectories, including trajectories with stable low and stable high symptoms of depression and at least two trajectories with intermediate symptoms with variations in slope (*intermediate increasing* and *intermediate decreasing*). A second aim was to analyze whether and to what extent sociodemographic variables that have been identified as risk factors for depressive symptoms (gender, family type, education, immigration, and economic deprivation) predict trajectory membership. The results of earlier studies led us to expect that female gender, lower level of education, economic deprivation, single-parent and stepfamily status, and immigration background would be associated more with trajectories of higher depressive symptoms with reference to trajectories with no, low, or decreasing depressive symptoms.

## Method

### Procedure and Design

Our analyses are based on Waves 2–8 of the pairfam study. A detailed description of the study has been provided by Huinink et al. (2011). The pairfam study started in 2008/2009 with a nationally representative sample of three birth cohorts (1971–1973, 1981–1983, and 1991–1993). A total of more than 12,000 computer-assisted personal interviews for all cohorts were conducted in pairfam’s Wave 1 (2008/2009), followed by annual reinterviews. For the present study, only the cohort born in 1991–1993 was considered, as adolescents and their subsequent course of development were our focus. The first wave was not included because depressive symptoms were assessed only from the second wave onward. All participants of the respective birth cohort who completed Waves 2 (15-19 years) until Wave 8 (21-25 years) were considered with a 30% tolerance of missing data (Collins et al., 2001). The sociodemographic predictors were selected according to their last assessment date.

### Participants

Participants were 3,682 adolescents and young adults who participated in the pairfam study (49.3% female). Their age ranged from 15 to 19 years in Wave 2, that is, T1 in our analyses (*M* = 17.03 years, *SD* = 0.88) with 96.8% of the sample being in late adolescence (16-18 years). At T1, 95.7% of the participants reported living with their parents, and 4.2% had already moved out (three missing values). Most participants were German natives (76.3%), with the remaining having some form of immigrant background (21.9%) or missing data (1.8%). At T1, 7.9% of the participants had left school without a degree or were currently enrolled in a lower-level secondary school (Hauptschule), 34.7% were enrolled in a middle-level secondary school (Realschule), 42.4% were enrolled in a higher level secondary school (Gymnasium), and 15.0% were enrolled in some other type of school. Thirty percent of the parents (Wave 2) had completed a lower education level, 40% a middle education level, and 30% a higher education level. At T1, 65.2% of the youth were living in nuclear families, 11.6% were living in stepfamilies, and 21.3% were living with a single parent (2% missing data). The mean value for economic deprivation was *M* = 2.3 (*SD* = 1.0; Range = 1-5) with a median of 2.

### Measures

#### Depressive Symptoms

Depressiveness was assessed using the trait scale of the adapted German version of the State-Trait Depression Scales (STDS; Krohne et al., 2002). The trait scale instructs participants to report how they ‘‘generally’’ feel by rating themselves on a four-point frequency scale (1 = *almost never*, 4 = *almost always*). The scales had good reliability for all measurement occasions (Cronbach’s α ranged from .83 to .90 across waves) and a good external validity (Krohne et al., 2002). A sum score of > 25 with a range of 0-45 is defined as a cutoff for clinically relevant depressive symptoms.

#### Family Type

Using young people’s information regarding their biological parents’ partnership and current household composition in Wave 5 adolescents’ family type was defined as nuclear (both biological parents living together), single parent (parents separated or one parent deceased, no new partner in the adolescent’s household), or stepfamily (parents separated or one parent deceased, new partner in the adolescent’s household).

#### Economic Deprivation

The Economic Deprivation Scale comprises three items indicating the size of the household budget for ordinary living expenses: 1) *We have enough money for everything we need*; 2) *We often have to forego something because we have to watch our budget*; 3) *We are mostly short of money*. The items were derived from the Economic Deprivation Scale described in (Schwarz et al., 1997) and assessed in Wave 4. The response format ranged from 1 (*not at all correct*) to 5 (*completely correct*). Higher scores reflect higher economic deprivation.

#### Migration Background

Young people and/or their parents who were born outside of Germany and had non-German nationality were considered immigrants (first or second generation). The item was assessed in Wave 2.

### Analytic Strategy and Model Building

Analyses were conducted in Mplus Version 7.0 (Muthén & Muthén, 2017) using full information maximum likelihood. We chose the modeling techniques of latent class growth analysis (LCGA) and growth mixture modeling (GMM), to capture heterogeneity of depressive symptom trajectories. The main difference between LCGA and GMM lies in the modeling of the variance for intercept and slope. In LCGA, a special form of GMM, the variance for intercept and slope within the classes is fixed at 0, which allows for fewer parameters to be estimated (Berlin et al., 2014). We estimated a series of models (see Supplemental Table 1) with up to six trajectories for each modeling technique (LCGA vs. GMM). For the selection of the most suitable model, we used multiple fit indices as well as prior empirical evidence (Schubert et al., 2017). The relevant fit indices included information criteria such as the Akaike information criterion (AIC), where better fitting models have a lower AIC. Further, we implemented likelihood ratio tests where we assessed the relative fit of the model by comparing it to structurally similar models with one less class. Entropy was used to assess distinctiveness of classes, where higher entropy signals higher distinctiveness and more accurate assignment of individuals to classes (Infurna & Grimm, 2018). Further analyses were performed with R 2022.07.1. Once latent classes were identified, multinomial logistic regression was used to determine whether and to what extend sociodemographic variables predict a trajectory membership (dependent variable). One category of the dependent variable is chosen as the reference category, and other categories are compared to the reference. To correct for multiple comparisons, *p*-values were adjusted using the Benjamini-Hochberg procedure with a False Discovery Rate (FDR) set at 10%. This threshold was chosen in line with common practice in exploratory and multivariate research, where a more liberal FDR is acceptable to increase sensitivity and avoid Type II errors (Benjamini & Hochberg, 1995; Storey & Tibshirani, 2003). The correction was applied across all analyses, including those reported in the Supplementary Materials.

## Results

### Trajectories of Depressive Symptoms

The model comparisons are shown in Table 1. The entropy values for the LCGA solutions were generally higher than those for the GMM solutions, which speaks for a better assignment of the individuals to the trajectories within the LCGA solutions.

**Table 1:** Model Comparisons for LCGA and GMM for Depressive Symptom Trajectories

Measure	Class 1	Class 2	Class 3	Class 4	Class 5	Class 6
Linear LCGA						
AIC	23,727.87	18,771.84	17,393.61	16,866.48	16,607.66	16,446.48
BIC	23,793.77	18,846.37	17,486.77	16,978.28	16,738.10	16,595.55
SSA-BIC	23,765.17	18,808.24	17,439.11	16,921.09	16,671.37	16,519.29
Entropy		0.82	0.71	0.68	0.68	0.65
LMR *p*		< .01	< .01	< .01	< .01	.16
Linear GMM						
AIC	16,839.03	16,250.47	16,072.48	16,037.59		
BIC	16,913.57	16,356.06	16,209.13	16,205.29		
SSA-BIC	16,875.44	16,302.04	16,139.22	16,119.50		
Entropy		0.45	0.49	0.51		
LMR *p*		< .01	< .01	.07		

*Note.* LCGA = Latent class growth analysis; GMM = growth mixture modeling; AIC = Akaike information criterion; BIC = Bayesian information criterion; SSA = sample size adjusted; LMRp = Lo Mendell Rubin likelihood ratio test-*p*-values.

On the other hand, the lower (= better) information criteria (AIC, Bayesian information criterion [BIC], and sample-size-adjusted BIC) within each class were obtained with GMM, which indicates a better model quality. The likelihood ratio tests show that the LCGA approach offers a five-class solution as optimal, whereas the GMM approach produces a three-class solution as most suitable. When analyzing these two models in more detail, only the LCGA solution identified the characteristic trajectories of very high and very low burden. In summary, the entropy and the consistency with previous empirical data support the five-class solution of the LCGA, so the classification of this model was used for further analyses.

**Figure 1:**
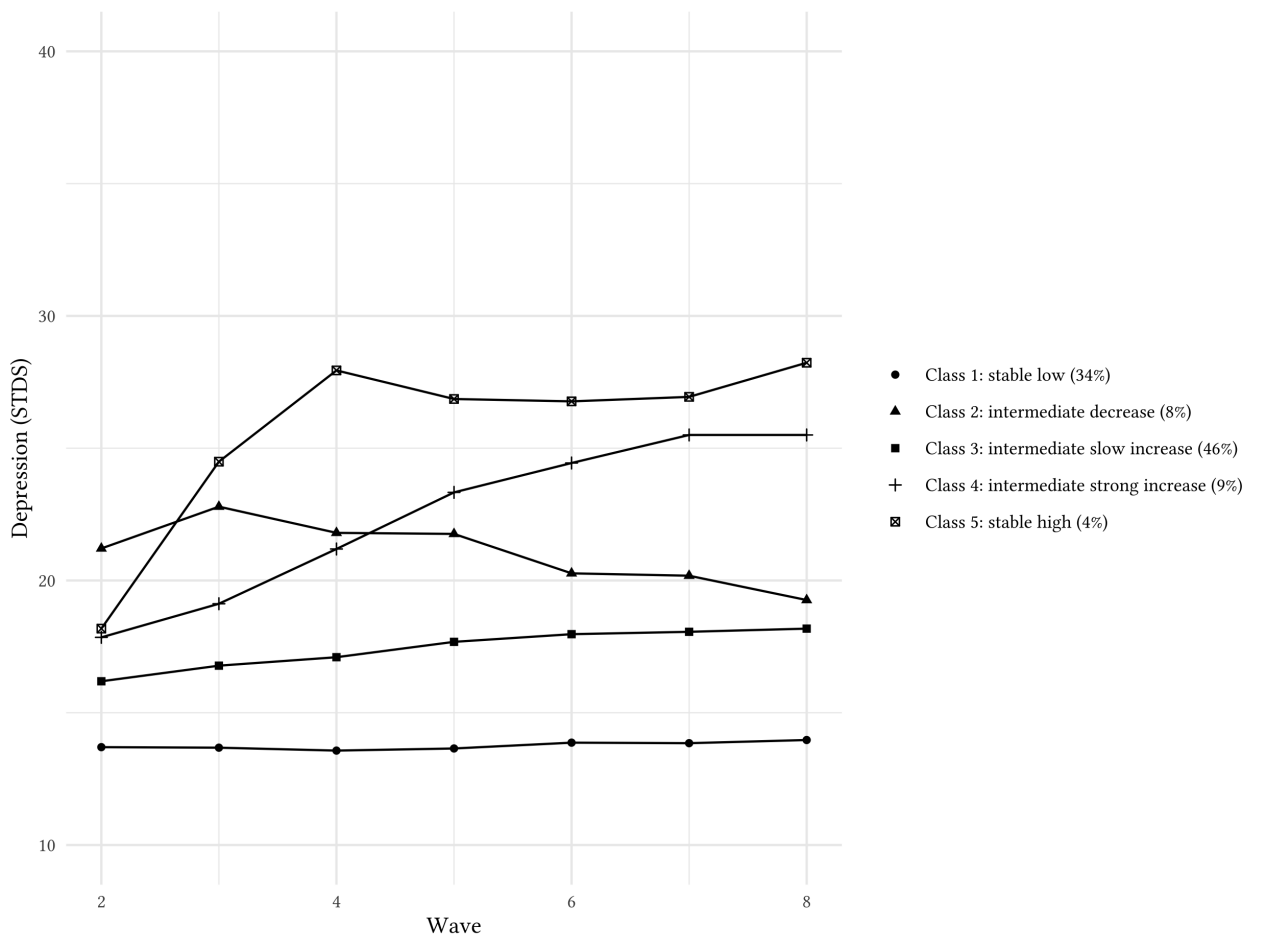
LCGA Model of Final Five-Class Solution With Size (%) of Each Class *Note.* STDS = State-Trait Depression Scales.

Figure 1 shows the final class solution with the associated gradients and the respective sizes of the classes. Class 1 comprises young people with stable low depressive symptoms (34%); Class 5 depicts the small group with stable high depressive symptoms (4%).

In addition, three classes have an intermediate starting point at T1, showing either a slight decrease (Class 2: 8%), a slow increase that levels off at the starting point of those with a strong increase (Class 3: 4%), or a strong increase across time that rises up to the level of the stable high group (Class 4: 9%), Class 2 has the highest starting point among all three intermediate groups and drops to a final level close to those with a slow increase.

### Predicting Membership in Trajectories by Sociodemographic Variables

Since economic deprivation is associated with family type and immigration status, we first calculated a model without economic deprivation as a predictor variable (see Supplemental Table 2). The group with stable low symptoms was used as a reference group. Female gender was a significant predictor for all four classes associated with depressive symptoms. Living in a stepfamily and having an immigration background were predictors for membership in the groups with strongly increasing symptoms and chronic trajectory. Living in a single-parent family was also a predictor for a chronic trajectory. Multicollinearity was low with a variance inflation factor (VIF) ranging from 1.64 to 4.27, with a model fit of AIC = 4,969.26.

Second, we included economic deprivation in the model with a better model fit of AIC = 3,764.56 and revealed high multicollinearity with a VIF of 9.90.

Table 2 reports the descriptive statistics of sociodemographic variables for each trajectory. The results of the four multinomial logistic regressions for all four models with odds ratios are reported in Table 3, 4, and 5.

**Table 2:** Descriptive Statistics of Sociodemographic Variables for Each Trajectory

Variable	Class 1 Stable low	Class 2 Intermediate decrease	Class 3 Intermediate slow increase	Class 4 Intermediate strong increase	Class 5 Stable high
***N***	1,241	292	1,678	317	154
**Gender (% female)**	40	60	50	58	73
Family type (%)					
Nuclear family	56	46	52	45	38
Stepfamily	26	28	31	31	33
Single-parent family	19	27	18	23	28
Parental education level (%)					
Lower	30	33	30	30	34
Middle	40	40	42	43	36
Higher	31	27	28	27	30
**Economic deprivation (*M*, *SD*)**	2.17 (0.99)	2.64 (1.07)	2.30 (0.98)	2.70 (1.14)	2.99 (1.09)
**Immigration background (%)**	19	28	20	25	27

**Table 3:** Multinomial Regressions Analyzing Sociodemographic Variables as Predictors of Trajectory Membership: Model 1

Variable	Model 1							
	Class 2 vs. Class 1		Class 3 vs. Class 1		Class 4 vs. Class 1		Class 5 vs. Class 1	
	*OR* [95% CI]	*p*	*OR* [95% CI]	*p*	*OR* [95% CI]	*p*	*OR* [95% CI]	*p*
**Intercept**	0.06 [0.03, 0.12]	< .001*	0.74 [0.51, 1.08]	.12	0.05 [0.03, 0.09]	< .001*	0.01 [0.00, 0.02]	< .001*
**Gender: female (ref. = male)**	2.70 [1.73, 4.20]	< .001*	1.47 [1.16, 1.86]	.01*	2.20 [1.52, 3.18]	< .001*	3.54 [1.97, 6.36]	< .001*
Family type (ref. = nuclear family)								
Stepfamily	0.99 [0.57, 1.72]	.98	1.27 [0.95, 1.69]	.10	1.30 [0.83, 2.01]	.25	1.24 [0.64, 2.38]	.52
Single-parent family	1.19 [0.64, 2.20]	.59	0.86 [0.60, 1.24]	.41	1.20 [0.71, 2.03]	.50	0.94 [0.42, 2.14]	.89
Parental education level (ref. = lower)								
Middle	0.63 [0.38, 1.07]	.09	1.08 [0.82, 1.43]	.57	1.16 [0.75, 1.79]	.50	1.16 [0.60, 2.22]	.66
Higher	0.87 [0.50, 1.51]	.62	1.07 [0.78, 1.45]	.68	1.05 [0.64, 1.74]	.83	1.19 [0.56, 2.53]	.64
**Economic deprivation**	1.45 [1.17, 1.80]	.001*	1.15 [1.01, 1.30]	.03*	1.43 [0.91, 2.25]	< .001*	2.08 [1.60, 2.70]	< .001*
**Immigration background (ref. = no immigration)**	1.32 [0.77, 2.25]	.31	1.28 [0.94, 1.74]	.12	1.69 [1.41, 2.02]	.12	1.64 [0.86, 3.14]	.13

*Note.* Class 1: stable low; Class 2: decrease; Class 3: slow increase; Class 4: strong increase; Class 5: stable high. CI = Confidence interval; *OR* = odds ratio.

*significant results for adjusted *p*-value thresholds.

In Model 1, female gender and higher economic deprivation were significant predictors for all four classes associated with higher depressive symptoms with reference to the class with stable low depressive symptoms. Education, family type, and immigration background were not associated with memberships in trajectory classes.

**Table 4:** Multinomial Regressions Analyzing Sociodemographic Variables as Predictors of Trajectory Membership: Model 2

Variable	Model 2					
	Class 3 vs. Class 2		Class 4 vs. Class 2		Class 5 vs. Class 2	
	*OR* [95% CI]	*p*	*OR* [95% CI]	*p*	*OR* [95% CI]	*p*
**Intercept**	12.70 [6.34, 25.43]	< .001*	0.82 [0.35, 1.91]	.65	0.12 [0.04, 0.39]	< .001*
**Gender: female (ref. = male)**	0.54 [0.35, 0.84]	.001*	0.82 [0.49, 1.36]	.43	1.31 [0.66, 2.60]	.43
Family type (ref. = nuclear family)						
Stepfamily	1.28 [0.75, 2.18]	.36	1.31 [0.70, 2.43]	.39	1.25 [0.57, 2.73]	.58
Single-parent family	0.72 [0.40, 1.32]	.29	1.01 [0.50, 2.04]	.97	0.80 [0.31, 2.02]	.63
Parental education level (ref. = lower)						
Middle	1.71 [1.03, 2.83]	.04	1.83 [1.01, 3.31]	.05	1.82 [0.84, 3.93]	.13
Higher	1.23 [0.72, 2.10]	.45	1.21 [0.63, 2.33]	.57	1.37 [0.58, 3.24]	.47
**Economic deprivation**	0.79 [0.64, 0.97]	.03*	1.16 [0.92, 1.48]	.22	1.43 [1.06, 1.94]	.02*
**Immigration background (ref. = no immigration)**	0.97 [0.58, 1.63]	.99	1.08 [0.59, 1.98]	.79	1.25 [0.59, 2.65]	.57

*Note.* Class 1: stable low; Class 2: decrease; Class 3: slow increase; Class 4: strong increase; Class 5: stable high. CI = Confidence interval; *OR* = odds ratio.

*significant results for adjusted *p*-value threshold.

In Model 2, female gender and economic deprivation were associated with a lower likelihood of a slow increase in depressive symptoms (Class 3) with reference to the class with decreasing symptoms (Class 2). Economic deprivation was a predictor for membership in the class with stable high symptoms (Class 5) with reference to the class with decreasing symptoms (Class 2).

In Model 3, female gender and economic deprivation were predictors for being in a trajectory with intermediate strong increasing symptoms (Class 4) with reference to the intermediate slow increasing trajectory (Class 3). In Model 4, odds ratios were non-significant for the trajectory with stable high symptoms (Class 5) with reference to the trajectory with strong increasing symptoms (Class 4).

**Table 5:** Multinomial Regressions Analyzing Sociodemographic Variables as Predictors of Trajectory Membership: Model 3 and 4

Variable	Model 3				Model 4	
	Class 4 vs. Class 3		Class 5 vs. Class 3		Class 5 vs. Class 4	
	*OR* [95% CI]	*p*	*OR* [95% CI]	*p*	*OR* [95% CI]	*p*
**Intercept**	0.06 [0.04, 0.12]	< .001*	0.01 [0.00, 0.03]	< .001*	0.15 [0.05, 0.44]	< .001*
**Gender: female (ref. = male)**	1.50 [1.05, 2.13]	.02*	2.42 [1.36, 4.29]	.001*	1.61 [0.85, 3.03]	.14
Family type (ref. = nuclear family)						
Stepfamily	1.02 [0.67, 1.55]	.93	0.97 [0.52, 1.84]	.94	0.96 [0.47, 1.94]	.90
Single-parent family	1.40 [0.84, 2.32]	.20	1.10 [0.49, 2.45]	.82	0.79 [0.33, 1.88]	.60
Parental education level (ref. = lower)						
Middle	1.07 [0.71, 1.62]	.75	1.07 [0.56, 2.02]	.84	1.00 [0.49, 2.02]	.99
Higher	0.99 [0.61, 1.60]	.96	1.12 [0.54, 2.34]	.77	1.13 [0.50, 2.57]	.77
**Economic deprivation**	1.47 [1.24, 1.74]	< .001*	1.81 [1.41, 2.34]	< .001*	1.23 [0.93, 1.63]	.14
**Immigration background (ref. = no immigration)**	1.12 [0.73, 1.71]	.60	1.29 [0.69, 2.41]	.42	1.15 [0.57, 2.30]	.65

*Note.* Class 3: slow increase; Class 4: strong increase; Class 5: stable high. CI = confidence interval; *OR* = odds ratio.

*significant results for adjusted *p*-value threshold.

## Discussion

As expected, LCGAs of longitudinal data on depressive symptoms revealed subgroups of different trajectories, which were in the range of three to six trajectories, as Schubert et al. (2017) review indicated. Specifically, we found five trajectories, including as expected one trajectory with participants who experienced only minimal depressive symptoms (stable low/absence of symptoms) and one trajectory of adolescents who suffered from chronically high depressive symptoms (stable high/chronic). Furthermore, we found, as expected, intermediate trajectories with less severe depressive symptoms, which changed across time (negative and positive slopes). When the five trajectories are sorted by size, almost half of the adolescents (46%) reported moderate depressive symptoms with a slow increase across time (”intermediate slow increase”); a third (34%) indicated stable absence or low depressive symptoms (stable low); almost every 10th young person (9%) experienced intermediate depressive symptoms with a strong increase (intermediate strong increase); a similar-sized group (8%) indicated intermediate depressive symptoms tending to decrease; and 4% experienced chronic depressive symptoms (stable high).

In contrast to our study, the majority of previous studies found four trajectories of depressive symptoms in community samples. However, these studies typically started assessments at a younger age, and studies with a starting point at early adolescence (10–14 years) tended to find fewer trajectories compared to studies assessing depressive symptoms between late adolescence and middle adulthood. Additionally, the prevalence of stable high depressive symptoms (4%) was marginally lower compared to other findings (5–15%), which was also the case for the absence of depressive symptoms (34%), which deviated from previous findings (40–55%; Schubert et al., 2017). There is also a great heterogeneity in studies analyzing trajectories of depressive symptoms regarding measures of depressive symptoms, age span, and sociodemographic variables (Schubert et al., 2017). Studies with six trajectories found a trajectory with an adult onset with severe symptoms, however the sample consisted of a larger age span in adulthood compared to our sample.

As expected, trajectories with higher depressive symptoms with reference to a trajectory with the absence of symptoms were associated with female gender, which is in line with previous research (Crockett et al., 2020; Edwards et al., 2014; Hargrove et al., 2020; Mezulis et al., 2014). Female gender was also a stronger predictor for the trajectory of intermediate symptoms with strong increase than for the trajectory of intermediate symptoms with slow increase. Those results are consistent with findings that by late adolescence girls are more likely to develop depression at an earlier onset than boys (Rohde et al., 2013), which might explain the stronger increase of depressive symptoms. In their integrative model to explain gender differences in the prevalence of depressive symptoms, (Hyde et al., 2008) emphasized the interaction between biological vulnerability (e.g., genetic vulnerability, hormonal changes in puberty), affective vulnerability (e.g., temperament), cognitive vulnerability (e.g., negative cognitive style, internalizing of gender stereotypes), and negative life events (e.g., sexual harassment), which were shown in a longitudinal study (Mezulis et al., 2014). Gender stereotypes and unequal gender norms are also associated with depressive symptoms among adolescents (Koenig et al., 2021) and should be considered as etiological factors for gender differences. There is also evidence that youth identifying with male gender report different symptoms (e.g., more attention deficits, anhedonia) compared to youth identifying as female (depressive mood, sleep problems), which might also be a factor in gender differences in depressive symptoms (Crockett et al., 2020). Female gender was also a stronger predictor for the trajectory of intermediate symptoms with strong increase than for the trajectory of intermediate symptoms with slow increase. Interestingly, we found that female participants were also more likely to be in the intermediate trajectory with decreasing symptoms with reference to the trajectory with slowly increasing symptoms. The results might indicate that adolescents and young adults with subclinical symptoms of depression who identify as female might have a higher probability of coping with symptoms compared to those who identify as male. One explanation might be that youth identifying as female have a higher probability of seeking help for mental health problems (Zwaanswijk et al., 2003), which in this case might contribute to a decrease in symptoms over time. Furthermore, biological vulnerability might have less impact on transitioning into adulthood (Hyde et al., 2008). Measures should target the reduction of female gender discrimination (e.g. strengthening legal protections, economic empowerment initiatives, cultural and awareness campaigns and political representation).

Exploratively we analyzed if family type was a predictor for trajectories with higher depression levels given that living in a single-parent family has been identified as a risk factor for the development of depressive symptoms (Björkenstam et al., 2017; Laukkanen et al., 2016). When economic deprivation was not included, family status was a predictor for trajectories with intermediate increasing or chronic depressive symptoms. However, this effect disappeared completely when economic deprivation was included as a predictor in the model. Overall, it appears that in our sample, family status and immigration status were not risk factors per se, but only if they were associated with higher economic deprivation. The results are consistent with evidence that family type is associated with variables that might be considered independently (e.g., parental communication, coping with parental separation, lower family income) and that are not valid for every nonnuclear family system. The results are consistent with evidence that a higher risk for depression and behavioral problems is associated with other factors (e.g., children’s problems adjusting to divorce-related circumstances rather than the family structure itself; Amato, 2000).

Our findings with regard to the role of parental education differed from expectations based on other research (Korhonen et al., 2017; Quesnel-Vallée & Taylor, 2012), as parental education was not a predictor for trajectories with higher depression levels. The more relevant predictor of trajectories with higher depressive symptoms was economic deprivation, perhaps mediating weak effects of educational resources. As expected, trajectories with higher depression levels were associated with higher economic deprivation (Costello et al., 2003; Wickrama et al., 2009). Participants with economic deprivation were also more likely to be in the group with stable high symptoms than decreasing symptoms, emphasizing the chronicity of stress levels in families with low income (Najman et al., 2010). They were also more likely to be in the group with intermediate strong increasing symptoms than in the group with intermediate slow increasing symptoms demonstrating the early impact of economic deprivation. There are multiple reasons why low family income is associated with higher psychopathology. Families with low income report higher stress levels and are more vulnerable to further stress events (e.g., not living in a safe environment) and traumatic events, and there are more limited parenting resources as they also have a higher burden of parental psychopathology (Butler, 2014). The chronicity of stress levels in families with low income was also visible when comparing trajectories with each other, as we found a higher association with the stable high trajectory than in the intermediate decreasing trajectory. There is also evidence that higher socio-economic status is a facilitator for the achievement of developmental milestones associated with transitioning into adulthood, such as educational attainment (Johnson & Reynolds, 2013) and lower socioeconomic status associated with higher resource deprivation such as less parental and financial support (Prince et al., 2018). The findings emphasize the need for further political and societal measures to reduce economic deprivation as a risk factor. Evidence based measures to reduce economic deprivation include the investment in education, expansion of social safety nets, implementing progressive taxation, investment in infrastructure and affordable health care (Banerjee & Duflo, 2011; OECD, 2015). Economic deprivation was also a predictor for a group membership in the intermediate decreasing symptom trajectory with reference to the trajectory with slow increasing symptoms. In addition to the reduction of risk factors, the strengthening of protective factors also plays an important role in coping with higher stress levels. Stable employment and financial security alleviate the stress associated with poverty, reducing the risk of depression in emerging adulthood (Murali & Oyebode, 2004).

The last sociodemographic variable we considered was young people’s immigration background, asking if it was associated with trajectories of higher depression levels. Comparable to family status, immigration was no longer a predictor when deprivation was included in the model. Overall, the findings suggest that it is not immigration per se that is a risk factor, but the circumstances associated with it, which should be addressed accordingly to prevent the development of depressive disorders.

### Limitations and Strengths

The person-centered approach based on longitudinal data of a community sample, which covered the time span from late adolescence to emerging adulthood, is one particular strength of our study. Although this approach represents a reduction in complexity, as measurement points of depressive symptoms were limited to a 1-year time span, there is evidence that depressive symptoms in adolescents remain rather stable over a 1-year time span (McLaughlin & King, 2015). LCGA is a powerful tool for identifying latent subpopulations (depressive symptom trajectories) within a sample according to patterns of responses to observed variables (Muthén & Muthén, 2017) but class assignment is based on probabilities and may neglect infrequent patterns in the course of depressive symptom. Trajectories might also vary according to methodological differences in study design. It can further be discussed whether the low-stable and intermediate-slow increase groups are in fact meaningfully different, as the differences between these two trajectory groups for gender and economic disadvantage are much smaller than for the comparisons between the low-stable group and the other groups. Overall, however, our findings are well in line with previous research and provide new evidence pertaining to the phase from adolescence to emerging adulthood, which represents an important time window of development. Another limitation is the use of the measurement instrument to assess depressive symptoms. A measure that enables greater comparability with other studies (e.g. CES-D) would have been of advantage. We assessed gender, family type, parental education, economic deprivation, and immigration background as predictors for depressive symptom trajectories, but there may be other predictors that were not considered in the current study but could be important in understanding the development of depressive symptoms. Examples include family support, school climate, peer belonging (Gregory et al., 2020), and—not least—treatment use. The analyzed predictors are also not independent of each other. The risk of poverty is higher for single parents and income depends on the level of education (OECD, 2024). The fact that we did not control for possible changes in the predictors (e.g., economic deprivation, family status) across the different waves is also a limitation of the study. Sociodemographic variables do not cover the complexity of risk factors as risks can arise from the interplay of multiple factors within the guise of the analyzed variables. The FDR rate of 10%, which was used for alpha error inflation instead of the more conservative rate of 5%, can also be discussed critically. Although links between trajectories of depressive symptoms and adolescents’ help-seeking behavior would be of great interest, such issues require a different strategy of data analysis and will be addressed in future analyses. The data analysis was not preregistered, which should be considered for future analysis.

### Conclusion

Understanding trajectories of depressive symptoms as well as risk and protective factors in the development of depressive disorders is crucial for identifying youths at risk for depression and increasing the probability of receiving adequate treatment. Longitudinal data on depressive symptoms in late adolescence transitioning into adulthood have demonstrated heterogeneous trajectories, enabling us to identify female gender and economic deprivation as risk factors for group membership in trajectories associated with higher and more stable levels of depressive symptoms. On an intermediate subclinical level of depressive symptoms, female gender was also associated with a higher probability of membership in a trajectory with decreasing symptoms over time than in one with increasing symptoms, suggesting more protective factors among females for coping with subclinical depressive symptoms. Family status and immigration status were not significant risk factors per se but were instead mediated by economic deprivation. It is of great importance to take measures to reduce female gender discrimination and economic deprivation as risk factors to mitigate the risks of developing depressive disorders. Such measures (e.g., political and societal initiatives) should target the underlying etiology of both constructs, taking into consideration the complexity and interaction of biopsychosocial variables. These results also emphasize the need for early detection and treatment of depressive symptoms, especially in the trajectories of stable high and strongly increasing symptoms.

## Supplementary Materials

The Supplementary Materials contain the following items (for access, see Pfeiffer et al., 2025S):

- **Supplement 1.** Results of LCGA and GMM Analyses for Depressive Symptom Trajectories This supplement provides detailed results of latent class growth analysis (LCGA) and growth mixture modeling (GMM) examining trajectories of depressive symptoms. For each model specification (linear, latent base course, quadratic, and LCGA linear), the supplement includes density plots, estimated trajectories, trajectories overlaid with raw data, and model fit indices (AIC, BIC, aBIC, entropy, VLMR/LMR *p*-values, and class sizes). These materials offer transparency regarding model selection and facilitate evaluation of alternative class solutions.
- **Supplemental Table 2.** Multinomial Regression Analyses of Trajectory Membership Predictors This table presents results from multinomial regression models examining sociodemographic variables (gender, family type, parental education, immigration background) and treatment use as predictors of depressive symptom trajectory membership. Odds ratios (*OR*), 95% confidence intervals (CI), and *p*-values are reported for each class comparison.

PfeifferS.
AltP.
WalperS.
 (2025S). Supplementary materials to "Trajectories of depressive symptoms and associated risk factors from late adolescence to emerging adulthood"
[Additional analyses and results]. PsychOpen. 10.23668/psycharchives.21282
PMC1292319741725701

## Data Availability

Data of the pairfam study are available at https://www.pairfam.de on request.

## Preregistration

This data analysis was not preregistered.

## Reporting Guidelines

This manuscript follows the *APA Journal Article Reporting Standards for Quantitative Research (JARS–Quant),* including the extended guidelines for longitudinal studies.
